# Variation in Uteroglobin-Related Protein 1 (*UGRP1*) gene is associated with Allergic Rhinitis in Singapore Chinese

**DOI:** 10.1186/1471-2350-12-39

**Published:** 2011-03-16

**Authors:** Anand Kumar Andiappan, Wei Sheng Yeo, Pallavi Nilkanth Parate, Ramani Anantharaman, Bani Kaur Suri, De Yun Wang, Fook Tim Chew

**Affiliations:** 1 Department of Biological Sciences, National University of Singapore, Science Drive 4, 117543, Singapore; 2 Department of Otolaryngology, National University of Singapore, 10 Lower Kent Ridge Road, 119260, Singapore

## Abstract

**Background:**

Uteroglobin-Related Protein 1 (*UGRP1*) is a secretoglobulin protein which has been suggested to play a role in lung inflammation and allergic diseases. UGRP1 has also been shown to be an important pneumoprotein, with diagnostic potential as a biomarker of lung damage. Previous genetic studies evaluating the association between variations on *UGRP1 *and allergic phenotypes have yielded mixed results. The aim of this present study was to identify genetic polymorphisms in *UGRP1 *and investigate if they were associated with asthma and allergic rhinitis in the Singapore Chinese population.

**Methods:**

Resequencing of the *UGRP1 *gene was conducted on 40 randomly selected individuals from Singapore of ethnic Chinese origin. The polymorphisms identified were then tagged and genotyped in a population of 1893 Singapore Chinese individuals. Genetic associations were evaluated in this population comparing 795 individuals with allergic rhinitis, 718 with asthma (of which 337 had both asthma and allergic rhinitis) and 717 healthy controls with no history of allergy or allergic diseases.

**Results:**

By resequencing the *UGRP1 *gene within our population, we identified 11 novel and 16 known single nucleotide polymorphisms (SNPs). TagSNPs were then genotyped, revealing a significant association between rs7726552 and allergic rhinitis (Odds Ratio: 0.81, 95% Confidence Interval: 0.66-0.98, P = 0.039). This association remained statistically significant when it was analyzed genotypically or when stratified according to haplotypes. When variations on *UGRP1 *were evaluated against asthma, no association was observed.

**Conclusion:**

This study documents the association between polymorphisms in *UGRP1 *and allergic rhinitis, suggesting a potential role in its pathogenesis.

## Background

Allergic diseases such as asthma, allergic rhinitis and atopic dermatitis are global health problems affecting 10-25% of the world's population. Allergic rhinitis (AR) is an IgE-mediated inflammatory disease of the nasal mucosa which is caused by exposure to allergens. AR is characterized by hyper-responsiveness, high levels of Th2 cells [1] and manifestation of symptoms such as rhinorrhea, sneezing, nasal congestion and rhino-conjunctivitis. AR affects approximately 500 million people worldwide [2,3]. In Singapore, the prevalence of AR was reported to be 13.1% [4]. AR is not a life threatening disease; however its impact on the quality of life and productivity is significant [3,5]. In addition, AR is known to be associated with other conditions such as asthma, sinusitis, anosmia, otitis media, nasal polyps, lower airway infections and dental malocclusion [2,6-8].

Many reports support a genetic basis for atopy and allergy [9,10]. The underlying pathogenic mechanism of allergic diseases is not fully elucidated and may be the result of complex interactions between genetic and environmental factors [2,6]. Studies looking at twins have provided convincing evidence for a genetic influence, as observed by the greater concordance of allergic manifestations in monozygotic compared to dizygotic twins [11-14]. Non-genetic factors such as an increase in exposure to irritants and allergens, changes in lifestyle, nutrition, pollution and stress, may also influence the onset and development of atopic diseases [7,15-17]. Individuals with a strong family history of allergic disease are more likely to develop allergic symptoms, irrespective of the varying environmental risk factors across societies [15,18].

The gene coding for secretoglobulin (*SCGB3A2*), also known as *UGRP1*, is located within chromosome 5q31-33. This region of the human chromosome 5 also contains many other candidate genes for allergic diseases such as interleukins - *IL-3*, *IL-4*, *IL-5*, *IL-9*, *IL-13*; macrophage colony stimulating factor (*CSF*) and β_2_-adrenergic receptor (*ADRB2*) [19]. Genetic variations in *UGRP1 *have been associated with autoimmune diseases such as Hashimoto thyroiditis (HT) and Graves' disease (GD) [20], and allergic diseases such as asthma [21]. A promoter polymorphism in the gene (G-112A) was found to increase the risk of asthma in a Japanese population [19]. However, the association was not replicated in studies involving Indians [22] and German Caucasians [23]. There has not been any association study evaluating the role of these polymorphisms in AR to date. In Singapore a family based linkage study had previously identified the 5q31-33 region to be significantly linked to atopy and asthma in the local ethnic Chinese population [24]. As part of a larger study to fine-map candidate genes for atopy and asthma in this chromosome region, resequencing of the *UGRP1 *gene was performed, with the aim of identifying novel polymorphisms in Singapore Chinese and evaluating their association to allergic rhinitis and asthma.

## Methods

### Ethics Statement

This study has been performed with the approval of the Institutional Review Board (IRB, Reference - NUS07-023 and NUS10-343) of the National University of Singapore and is in compliance with the Helsinki declaration.

### Study Population

Ethnic Chinese subjects were recruited at the National University of Singapore, KK Women's and Children Hospital and the National University Hospital as a part of an on-going epidemiological collection for the study of allergic diseases and through multiple recruitment drives in Singapore (Table 1). Those not born in Singapore and did not reside locally in the past 10 years were excluded. Volunteers were then classified as individuals with AR, asthma and healthy controls according to their status as determined by an interviewer-administered questionnaire based on the Allergic Rhinitis Impact on Asthma (ARIA) and International Study of Asthma and Allergies in Childhood (ISAAC) guidelines [2,6], and a doctor's diagnosis. The validity and use of ARIA- and ISAAC-based questionnaires has been discussed by others groups [25,26] and ours as well [27]. In addition, all volunteers were subjected to a skin prick test (SPT) using a panel consisting of common allergens in Singapore such as *Dermatophagoides pteronyssinus *(house dust mite), *Blomia tropicalis *(dust mite), *Elaeis guineensis *(pollen) and *Curvularia lunata *(fungi). A SPT response is considered positive when the wheal diameter is 3 mm or greater, when compared to positive (histamine) and negative (saline) controls. The selection of these allergens was based on previous studies in the region which clearly demonstrated that sensitization of the major indoor allergen - the house dust mite (HDM), was considered an important risk factor for the development of asthma and allergic diseases, especially in South East Asian populations, such as those from Singapore, Malaysia and Thailand [28-30]. Based on our previous epidemiological studies [31,32], the majority of AR and atopic asthma individuals would be sensitized to one or both of the major dust mites evaluated (*Dermatophagoides pteronyssinus*, and/or *Blomia tropicalis*) and up to 30% would be poly-sensitized (to dust mites and pollen and/or fungal allergens). Atopy was defined as a positive SPT response to either dust mite allergen extracts, with or without poly-sensitization to other allergens. AR was diagnosed based on the presence of the atopic status and typical AR symptoms as defined by the ARIA 2008 guidelines [2,6], i.e., two or more AR symptoms (nasal congestion, rhinorrhea, nasal itching, sneezing) persisting for four or more days a week during the past year. Asthma was classified based on a positive doctor-diagnosed asthma with wheezing symptoms and positive skin prick responses to common allergens. A subset of the asthmatic individuals would have complete ACT (Asthma Control Test) scores, lung function and peak flow reversibility data. (This is however currently on-going, and thus not reported in this study). Conversely, healthy control individuals were defined as those with no atopy (i.e., skin prick negative) and with no history of allergic conditions, diagnosis, or symptoms.

**Table 1:** Demographic and clinical characteristics of the samples used in the study

	**Asthma**^**#**^	**Allergic Rhinitis (AR)**^**$**^	**Healthy controls**^^^
***Subjects***	718	795	717

***Age, mean***	20.24	21.06	22

***Gender***			

***Male***	377 (52.5%)	385 (48.4%)	196 (27.3%)

***Female***	341 (47.5%)	410 (51.6%)	521 (72.7%)

^# ^an asthma case was classified based on doctor diagnosis of the disease with a positive skin prick test reaction to one of the allergens tested *(based on WHO and GINA guidelines for asthma)*.

^$ ^Allergic Rhinitis (AR) was classified based on 2 or more major symptoms which include (nasal congestion, rhinorrhea, nasal itching, sneezing) and a positive skin prick test reaction to one of the allergens tested *(based on 2008 guidelines set by Allergic Rhinitis Impact on Asthma (ARIA) consortium).*

^^^Healthy controls are individuals classified based on no symptoms or history of allergic disease and are non- atopic (Negative skin prick test reaction to ALL of the allergens).

Note: All samples have been age-category matched.

### Resequencing

Genomic DNA was extracted from buccal cells obtained from 15 ml of mouthwash in 0.9% saline solution as previously described [33]. DNA was then quantified using Nanodrop and by flourimetric analysis using Pico-Green (Molecular Probes, Invitrogen, OR, USA). DNA from a subset of 40 randomly selected individuals was used for sequencing and the identification of genetic polymorphisms [34]. The 3 exons and exon-intron boundaries of *UGRP1 *were sequenced (Additional File 1). Information on primer sequences and the regions covered by resequencing have been described below in Additional File 2.

### Genotyping

Genotyping was performed on the Illumina BeadXpress platform (Illumina Inc., San Diego, CA, USA) at the University of Utah Genomics Core Facility (Salt Lake City, UT, USA) according to manufacturer's recommendations.

### Statistical Analysis

Linkage Disequilibrium (LD) blocks were generated with the SNPs identified using Haploview v4.2 [26]. Hardy-Weinberg equilibrium (HWE) was assessed in the control population as a quality control measure. Tests for association were performed at the allelic, genotypic and haplotypic levels and odds ratio with 95% confidence intervals were estimated by PLINK v1.06. A *P*-value less than 0.05 were considered statistically significant (with Bonferroni corrections for multiple testing where necessary).

### *In silico *analysis using bioinformatics tools

*In silico *analysis was performed using bioinformatics tools such as TRANSFAC, http://www.gene-regulation.com/cgi-bin/pub/databases/transfac/search.cgi and TFSEARCH http://www.cbrc.jp/research/db/TFSEARCH.html to predict potential transcription factor binding site (TFBS) and possible effects of the polymorphisms on the binding of transcription factors. The results were cross compared using another transcription factor prediction server, ALIBABA v2.1 http://www.gene-regulation.com/pub/programs/alibaba2/index.html.

## Results

### Resequencing

**Figure 1:**
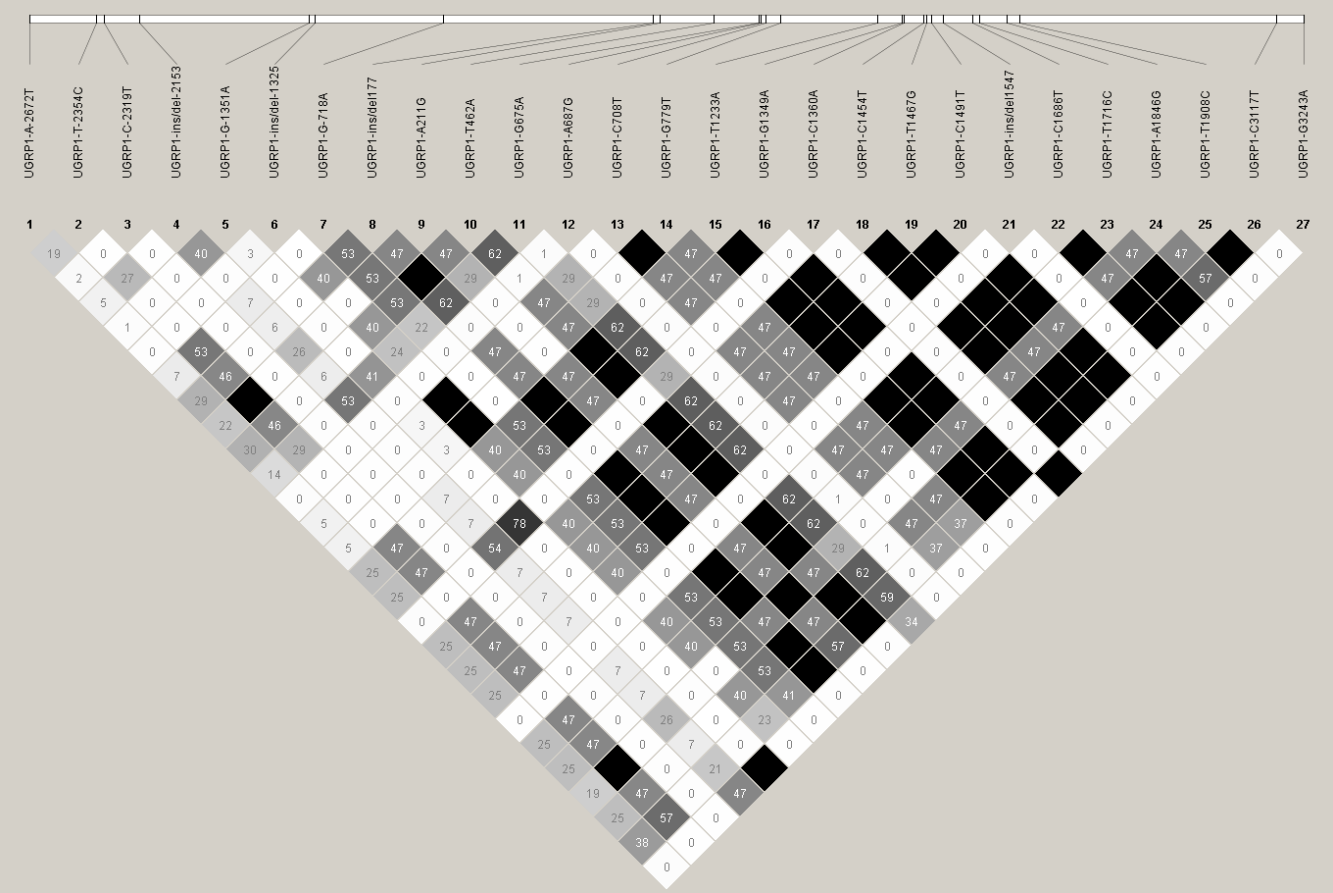
**LD block of the Singapore Chinese population for the *UGRP1 *gene**. Linkage disequilibrium block generated using SNPs identified in the *UGRP1 *gene. Figure generated using Haploview 4.1 and the values denoted in the boxes represent R^2 ^values which are a measure of the linkage between SNP pairs.

Of the 27 polymorphisms identified through resequencing of the *UGRP1 *gene [23 SNPs and 4 INDELs (INsertion/DELetion)], 11 were novel (Additional File 3). Of the 16 polymorphisms previously reported in NCBI, only 4 SNPs were present in the HapMap database for the Han Chinese (CHB) population. LD blocks were constructed using r^2 ^values estimated for all SNPs identified through the resequencing of the *UGRP1 *gene (Figure 1). Tagging was performed using an LD threshold of r^2 ^= 0.8 resulting in a total of 11 tagSNPs representing the variations in *UGRP1 *among Singapore Chinese. (Additional File 4)

### Single SNP marker association analysis

**Figure 2:**
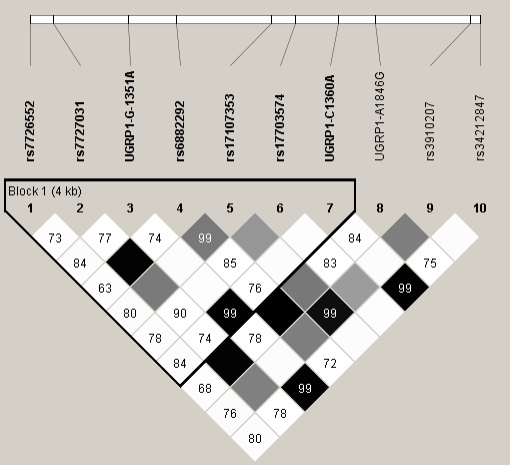
**LD block generated using tagSNPs genotyped for the *UGRP1 *gene**. Linkage disequilibrium block generated using tagSNPs genotyped for the entire study population. Figure generated using Haploview 4.1 and the values denoted in the boxes represent R^2 ^values which are a measure of the linkage between SNP pairs.

A total of 10 out of the 11 tag SNPs in *UGRP1* (Figure 2), targeted for genotyping, passed the BeadXpress assay design quality control and were successfully genotyped in 1893 samples (with a genotyping success rate of 99.6%). Allele based association analyzed using the Cochran Armitage trend test for the AR phenotype revealed that rs7726552 is significantly associated (*P*_*trend *_= 0.032, OR = 0.81) (Table 2). Association at the genotype level appeared stronger with *P*_*genotype *_= 0.00085 and OR = 0.79 (*P*_Bonferroni _= 0.0085) (Table 3). No significant association was detected for the asthma phenotype (Additional File 5).

**Table 2:** Allele based association test for Allergic Rhinitis (AR) phenotype

SNP	Position	Minor allele	Allele Frequency		Major Allele	**P**_**trend**_	OR	L95	U95
			Cases	Controls					
rs7726552	147235795	T	0.14	0.17	A	0.039*	0.81	0.66	0.98

rs7727031	147236113	C	0.06	0.06	T	0.544	0.91	0.67	1.23

*UGRP1*-G-1351A	147237116	A	0.07	0.07	G	0.641	1.07	0.81	1.41

rs6882292	147237749	A	0.06	0.06	G	0.739	0.95	0.70	1.28

rs17107353	147239022	A	0.11	0.12	T	0.154	0.85	0.67	1.06

rs17703574	147239339	T	0.05	0.06	G	0.141	0.79	0.56	1.08

*UGRP1*-C1360A	147239920	A	0.07	0.07	C	0.81	1.04	0.78	1.36

*UGRP1*-A1846G	147240405	G	0.06	0.06	A	0.687	0.94	0.69	1.27

rs3910207	147241677	T	0.11	0.13	C	0.202	0.87	0.69	1.08

rs34212847	147241803	A	0.07	0.07	G	0.668	1.06	0.80	1.4

P_trend _is calculated by the Cochran Armitage trend test using the PLINK v 1.06 software.

* P_trend _< 0.05 for the association test for AR.

OR - Odds ratio; CI - Confidence Interval.

**Table 3:** Genotype based association test for Allergic Rhinitis (AR) phenotype

SNP id	Minor Allele	Major Allele	Model	Genotype	Case (%)	Control (%)	**χ**^**2**^	P
								

rs7726552	T	A	**Full genotype**	TT	16 (2.02)	6 (0.84)		
		
				TA	190 (23.93)	227 (31.70)		
		
				AA	588 (74.06)	483 (67.46)	7.95	0.00085*

								

			**Dominant**	TT+TA	206 (25.94)	233 (32.54)		
		
				AA	588 (74.06)	483 (67.46)	14.13	0.0048^#^

* P value calculated using χ^2 ^test of association for all the three genotypes.

^# ^P value calculated using χ^2 ^test of association for a dominant model.

### Haplotype based association analysis

Haplotypes within *UGRP1 *were tested for significant associations with the atopic conditions using PLINK. The haplotype H2 (ATGGTGC), covering a region of 4.13 kb, encompassing the promoter and the first intron, and consisted of the SNPs rs7726552, rs7727031, *UGRP1*-G-1351A, rs6882292, rs17107353, rs17703574 and *UGRP1*-C1360A, was found to be the most common with a frequency of 69.6% in controls and 65.5% in cases (P = 0.017) (Table 4). These results are consistent with the single SNP association of rs7726552 with AR, and where the allele frequencies were 14% in cases and 17% in controls (P = 0.039).

**Table 4:** Association of AR associated haplotypes in *UGRP1 *gene

Haplotype	rs7726552	rs7727031	*UGRP1*-G-1351A	rs6882292	rs17107353	rs17703574	*UGRP1*-C1360A	Haplotype frequency		P value*
									
								717 controls	795 cases	
H1	A	C	G	A	A	G	C	0.053	0.059	0.536

H2	A	T	G	G	T	G	C	0.696	0.655	**0.017**

H3	A	T	A	G	T	G	A	0.071	0.066	0.637

H4	T	T	G	G	T	G	C	0.135	0.162	**0.036**

H5	A	T	G	G	A	T	C	0.046	0.058	0.120

Haplotypes are constructed using the sliding window method by PLINK software. Haplotype frequencies are estimated using the Haploview software.

* P value calculated using χ^2 ^test of association for haplotypes.

### *In silico *prediction of putative function

TRANSFAC was used to predict possible TFBS which might be affected due to the SNP. The *in silico *prediction tool revealed that the region containing SNP rs7726552 has potential consensus binding sites for transcription factors Oct-1, NF-1 and GATA-1. The A to T polymorphism was predicted to result in a new binding site for GATA-1, the loss of binding site for Oct-1 and no change in transcription factor binding for NF-1. Similar results were also shown using TFBS software ALIBABA 2.1.

## Discussion

UGRP1 was originally suggested to have an anti-inflammatory function due to its similarity to uteroglobin/Clara cell secretory protein [21]. *UGRP1 *is localized in the chromosome 5q31-32 region where asthma susceptibility locus has been assigned [35] and the high expression of this protein in epithelial cells of the airway [21] suggests a possible role in allergic airway inflammation. Further evidence of the role of UGRP1 is seen in its regulation by various T-regulatory cytokines such as IL-10 [36], IL-5 and IL-9 [37,38], suggesting its involvement in allergic response. Claire *et al.*, have shown that there is an increase in UGRP1 in the induced sputum of patients with asthma and rhinitis, further suggesting a possible role in inflammatory diseases [39].

The SNP rs7726552 showed significant association with AR in our population. Haplotype analysis revealed a single haplotype block which, when tested for association, was significantly different in cases as compared to controls. This haplotype block, a 4.13 kb region, includes the 5' upstream, promoter and the first intron. The SNP rs7726552 is present in the 5' upstream region and hence could affect the regulation of gene expression levels. Previous reports have shown that such polymorphisms in the *UGRP1 *gene could predispose an individual to allergic inflammation by reducing the levels of UGRP1 in the airway epithelial cells [19]. Interestingly, the promoter polymorphism G-122A, which was previously associated with asthma in Japanese people [19] was also identified in our population and was designated as *UGRP1*-G-1351A. However, similar to studies on Indians and German Caucasians, the association to asthma for this SNP was not replicated in the Singapore Chinese population. This could be attributed to underlying differences in the genetic makeup of the populations evaluated. The evaluation of the influence of rs7726552 on UGRP1 expression and regulation would be helpful in identifying the role it may play in allergy. Much is known as to how a gene is regulated and this may involve multiple potential mechanisms such as differential gene splicing or binding of transcription factors to regulatory elements. The presence of SNPs in these regulatory regions might then predispose an individual to disease [40,41]. The *in silico *analysis revealed potential binding sites for Oct-1, NF-1 and GATA-1 at the 5' upstream locus where rs7726552 was identified. Oct-1 has been previously shown to be important in regulating the expression of *IL13 *which is a key regulator of Th2-mediated inflammation in allergic diseases. Kiesler *et al.*, demonstrated that a polymorphism in the regulatory element affects the transcription of the *IL13 *gene by creating a binding site for Oct-1 [42]. Similarly, Hasegawa *et al.*, demonstrated that the T allele on the Fc epsilon RI alpha-chain promoter introduced an additional binding motif for GATA-1 compared to the C allele and hence the transcription activity of the T allele was enhanced because of the higher affinity for the transcription factor [43]. In a similar fashion, the A to T change in rs7726552 is predicted to result in the loss of the binding site for Oct-1 and conversely introduce an additional binding site for GATA-1. This prediction suggests why the T allele confers protection (OR 0.81) with a higher proportion of the controls (17%) having the allele as compared to the cases (14%). The genotyping results showed more evidence for a dominant model of association producing a P_dominant _= 0.0048 and OR = 0.79. The functional significance and the mechanism of the causal variant in *UGRP1 *leading to the onset of allergic rhinitis needs to be confirmed and validated with further studies.

Haplotype association analysis revealed a common haplotype on *UGRP1 *to have significant different frequencies between cases and controls. This haplotype block contains 7 SNPs from the promoter region and first intron. Previous reports on the *UGRP1 *gene have also confirmed the functionality of haplotype to be associated to the promoter region [36] and the regulation of *UGRP1 *expression in asthmatic patients [19]. Srisodsai *et al.*, have shown that in a mouse model, administration of IL10 increased constitutive *UGRP1 *mRNA expression and suggested UGRP1 as a potential target for IL-10 anti-inflammatory activities in the lung [36]. Chiba *et al.*, demonstrated that UGRP1 can suppress inflammation in a mouse model for allergic airway inflammation and also proposed UGRP1 as a therapeutic candidate for treating lung inflammation [35]. These reports highlight a potential role for UGRP1 in treatment of allergic airway diseases.

## Conclusion

In summary, our study reveals the association of *UGRP1 *polymorphisms, specifically rs7726552, with allergic rhinitis in the Singapore Chinese population. Haplotype analysis suggests that multiple polymorphisms in the gene could be contributing collectively to the pathogenesis of AR. Further functional characterization of these variants would be important in determining if these variants could be used as risk factors for AR.

## Competing interests

The authors declare that they have no competing interests.

## Authors' contributions

AKA, WSY, RA, PNP and BKS were involved in the design of the study, recruitment of participants for the study, and extraction of DNA samples. AKA, WSY performed the statistical analysis and drafted the manuscript. PNP performed the resequencing. DYW edited the manuscript. FTC conceived designed and planned the study, as well as edited the manuscript. All authors have read and approved the final manuscript.

## Pre-publication history

The pre-publication history for this paper can be accessed here:

http://www.biomedcentral.com/1471-2350/12/39/prepub

## Supplementary Material

Additional file 1 **Gene structure of *UGRP1***. Figure describing the structure of the UGRP1 gene on chromosome 5. Click here for file

Additional file 2 **Primers used for sequencing *UGRP1 *gene**. The list of primers used for sequencing of the UGRP1 gene Click here for file

Additional file 3 **Summary of polymorphisms identified through sequencing of *UGRP1 *gene**. Table summarizing the total polymorphisms identified by sequencing the UGRP1 gene. Click here for file

Additional file 4 **TagSNPs chosen for genotyping for case control association**. The list of SNPs selected for genotyping of the UGRP1 using the tagging approach. Click here for file

Additional file 5 **Association of *UGRP1 *SNPs to Asthma phenotype (Allele based test)**. Table summarizing the Association of the UGRP1 SNPs calculated using the allele based test for the asthma phenotype. Click here for file
