# Supplementary figures and images for: Variation in Uteroglobin-Related Protein 1 (UGRP1) gene is associated with Allergic Rhinitis in Singapore Chinese
Source: BMC Med Genet. 2011 Mar 16;12:39. doi: 10.1186/1471-2350-12-39 (PMC3070627; doi:10.1186/1471-2350-12-39)

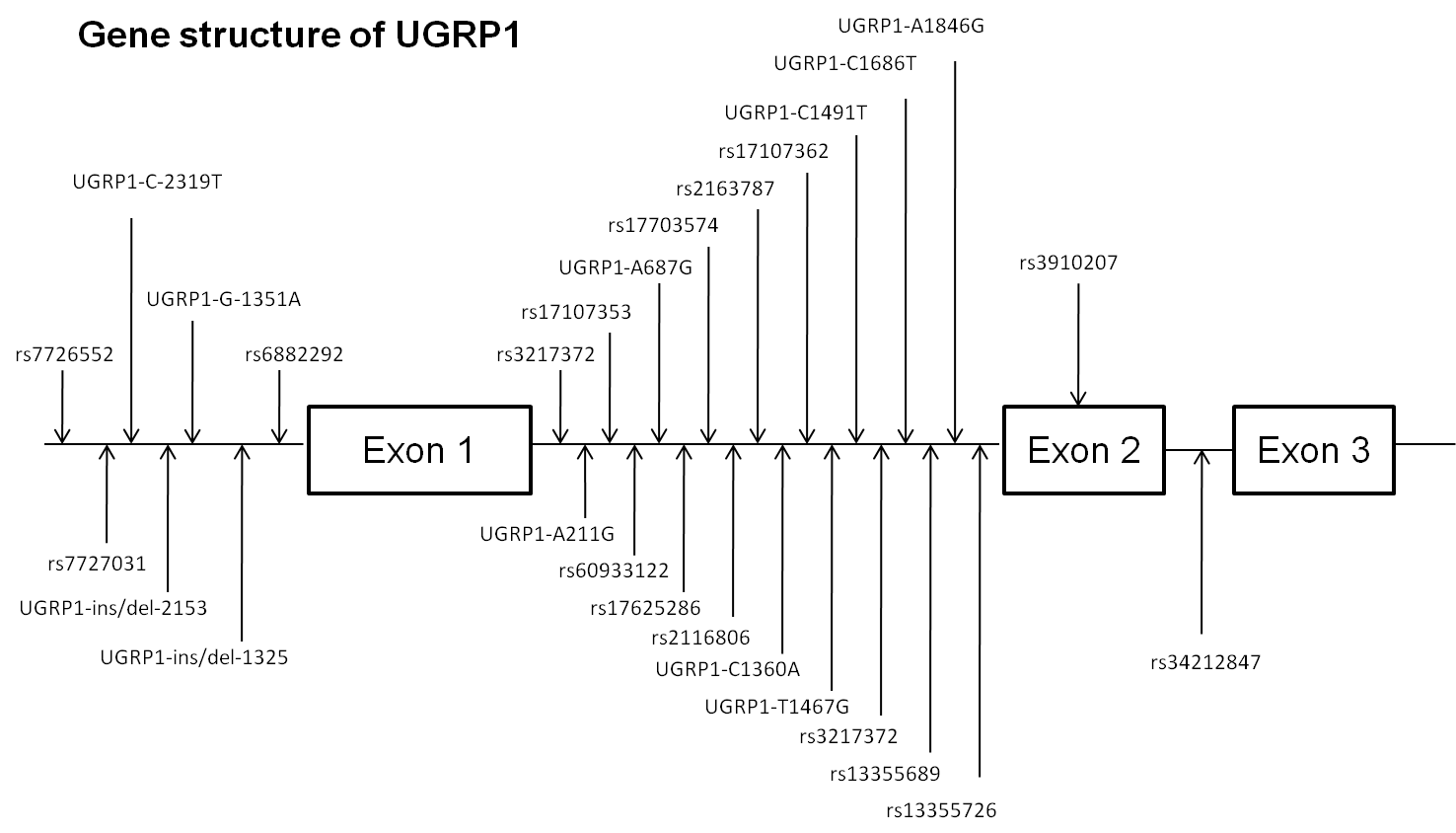

Supplement: Additional file 1 — Gene structure of UGRP1. Figure describing the structure of the UGRP1 gene on chromosome 5. [file 1471-2350-12-39-S1.BMP]
