# Supplementary material for: Variation in Uteroglobin-Related Protein 1 (UGRP1) gene is associated with Allergic Rhinitis in Singapore Chinese
Source: BMC Med Genet. 2011 Mar 16;12:39. doi: 10.1186/1471-2350-12-39 (PMC3070627; doi:10.1186/1471-2350-12-39)
Supplement: Additional file 2 — Primers used for sequencing UGRP1 gene. The list of primers used for sequencing of the UGRP1 gene [file 1471-2350-12-39-S2.DOC]

**Supplementary Table 1**: Primers used and regions resequenced on the UGRP1 gene

| **Sequencing primer** | **Primer Sequence 5'-3'** | **T_m_** | **Region Sequenced**  Start End | |
| --- | --- | --- | --- | --- |
| UGRP1-1F | GACCAGCAGAGAAGCCTGATACAGTACC | 64 | 147235665 | 147236800 |
| UGRP1-1R | AAATGAATTTCAAGCTCTATGCCTATATGC |  | 5’ upstream | |
| UGRP1-2F | ATCTTACCACCAACCTTGGCTCTTTGA | 60 | 147236972 | 147238034 |
| UGRP1-2R | AGCTTCATGACATTTAGTGCCACATTTC |  | 5’ upstream | |
| UGRP1-3F | AGAGGTACTTGAGAATGCTGTACTGTAGAG | 60 | 147242687 | 147243439 |
| UGRP1-3F | AATGAGCAGTGGCTATCATGAAATCT |  | Exon 1 | |
| UGRP1-4F | TCTCTAATGTCCAGGGACAGGTATCTAT | 59 | 147243693 | 147244488 |
| UGRP1-4R | GCATTCAACAGATCATTGTGATTGTCT |  | Intron 1 | |
| UGRP1-5F | AGACAATCACAATGATCTGTTGAATGC | 60 | 147245177 | 147246003 |
| UGRP1-5R | CTAATCTGGATACGTCCTTCTGAATAGAGT |  | Exon 2 and Exon 3 | |
|  | | | | |

*All Primers were purchased from 1^st^ base Asia Pvt. Ltd.*
