# Supplementary material for: Variation in Uteroglobin-Related Protein 1 (UGRP1) gene is associated with Allergic Rhinitis in Singapore Chinese
Source: BMC Med Genet. 2011 Mar 16;12:39. doi: 10.1186/1471-2350-12-39 (PMC3070627; doi:10.1186/1471-2350-12-39)
Supplement: Additional file 3 — Summary of polymorphisms identified through sequencing of UGRP1 gene. Table summarizing the total polymorphisms identified by sequencing the UGRP1 gene. [file 1471-2350-12-39-S3.DOC]

| **Study Reference ID** | **Type** | **Sequence of Polymorphism** | **Gene Position** | **Chromosomal position** | **rs id (NCBI)** |
| --- | --- | --- | --- | --- | --- |
| UGRP1-G-1351A | SNP | GGCAGGCATTCATG**(G/A)**TGTCTTT | 5'UTR | 147237116 | Not reported |
| UGRP1-ins/del-1325 | Indel | TTTATAAAGTA**(ins/del)** | 5'UTR | 147237142 | Not reported |
| UGRP1-G-718A | SNP | GTTATTTAT**(G/A)**TTCCCATTTT | 5'UTR | 147237749 | rs6882292 |
| UGRP1-G779T | SNP | AAGCCAGCTTCTATA**(C/A)**CCTTACC | Intron | 147239339 | rs17703574 |
| UGRP1-C708T | SNP | GGATATCTCACAGTAC**(G/A)**TTTACA | Intron | 147239268 | rs17625286 |
| UGRP1-A687G | SNP | TTTACAGTAGTCTAGAATTA**(T/C)**AGTAA | Intron | 147239247 | Not reported |
| UGRP1-G675A | SNP | AATTATAGTAACTTAGG**(C/T)**AGAAGCCC | Intron | 147239235 | rs60933122 |
| UGRP1-T462A | SNP | AAGAATGACTCCATAT**(A/T)**GTTCAAAA | Intron | 147239022 | rs17107353 |
| UGRP1-A211G | SNP | CCAACCCTGCAAATA**(T/C)**GTGCAATT | Intron | 147238771 | Not reported |
| UGRP1-ins/del177 | Indel | TTAAAAGTAAAAT**(ins/del)** | Intron | 147238738 | rs3217372 |
| UGRP1-T1233A | SNP | GATTGAAAATT**(T/A)**AAAAAAATATATACA | Intron | 147239793 | rs2116806 |
| UGRP1-G1349A | SNP | ATGTGTGTTCTTACGTGT**(G/A)**TGCTCACA | Intron | 147239909 | rs2163787 |
| UGRP1-C1360A | SNP | GTGTRTGCTCACATG**(C/A)**ATGTGTGCA | Intron | 147239920 | Not reported |
| UGRP1-C1454T | SNP | CCCACCATTCATCTTCTA**(C/T)**GGGAAA | intron | 147240014 | rs17107362 |
| UGRP1-T1467G | SNP | GGGAAAGTTATC**(T/G)**TCCTAAA | Intron | 147240027 | Not reported |
| UGRP1-C1491T | SNP | AAAGATATAAAA**(C/T)**GTAATTTT | Intron | 147240050 | Not reported |
| UGRP1-ins/del1547 | Indel | CCCTCAGGAAAAAAAAA**(ins/del)** | Intron | 147240107 | rs3217372 |
| UGRP1-C1686T | SNP | TTCTGGGCAGAAGGCT**(C/T)**ACCATCC | Intron | 147240245 | Not reported |
| UGRP1-T1716C | SNP | GCACTTTTATCCTAA**(T/C)**GGTTCC | Intron | 147240275 | rs13355689 |
| UGRP1-A1846G | SNP | CAGCACTCTTTCATGTT**(A/G)**GAATTAG | Intron | 147240405 | Not reported |
| UGRP1-T1908C | SNP | TGTTAGCATAGCACA**(T/C)**GCTACACTGTGG | Intron | 147240467 | rs13355726 |
| UGRP1-G3243A | SNP | TTTATCTTGATGTCA**(C/T)**ACCAAGTGTC | Intron | 147241803 | rs34212847 |
| UGRP1-C3117T | SNP | TTTTACTGATG**(G/A)**GGAAACAAAAACTC | Coding | 147241677 | rs3910207 |
| UGRP1-A-2672T | SNP | TTTATAACTTGGC**(A/T)**TTTATAAGAA | 5'UTR | 147235795 | rs7726552 |
| UGRP1-T-2354C | SNP | AACAATGTTGGCCTG**(T/C)**GTGGCACAGGGG | 5'UTR | 147236113 | rs7727031 |
| UGRP1-C-2319T | SNP | TAAAGGACCTTT**(C/T)**CAAAAGGAGATGA | 5'UTR | 147236148 | Not reported |
| UGRP1-ins/del-2153 | Indel | GGAAGCCTGGAA**(ins/del)** | 5'UTR | 147236314 | Not reported |

**Supplementary Table 2: Polymorphisms identified through resequencing of the UGRP1 gene**
