# Supplementary material for: Variation in Uteroglobin-Related Protein 1 (UGRP1) gene is associated with Allergic Rhinitis in Singapore Chinese
Source: BMC Med Genet. 2011 Mar 16;12:39. doi: 10.1186/1471-2350-12-39 (PMC3070627; doi:10.1186/1471-2350-12-39)
Supplement: Additional file 4 — TagSNPs chosen for genotyping for case control association. The list of SNPs selected for genotyping of the UGRP1 using the tagging approach. [file 1471-2350-12-39-S4.DOC]

| **Tag SNP genotyped** | **Other SNPs captured in the same bin**  (r^2^>0.8 with the tagSNP) |
| --- | --- |
| UGRP1-A1846G  (rs7727031) | UGRP1-T-2354C, UGRP1-A211G |
| UGRP1-C2319T | N.A |
| rs6882292 | UGRP1-A-2672T |
| UGRP1-ins/del-2153 | N.A |
| rs6882292 | N.A |
| UGRP1-G-1351A  (rs34212847) | UGRP1-G3243A |
| UGRP1-C1360A | N.A |
| rs17703574 | UGRP1-ins/del-1325, UGRP1-C708T, UGRP1-G779T |
| rs17107353  rs3910207 | UGRP1-ins/del177, UGRP1-T462A, UGRP1-T1233A, UGRP1-G1349A, UGRP1-C1454T, UGRP1-T1467G, UGRP1-C1491T, UGRP1-C1686T, UGRP1-11716C, UGRP1-T1908C, UGRP1-C3117T* |
| UGRP1-ins/del1547 | UGRP1-ins/del1547 |
