# Supplementary material for: Variation in Uteroglobin-Related Protein 1 (UGRP1) gene is associated with Allergic Rhinitis in Singapore Chinese
Source: BMC Med Genet. 2011 Mar 16;12:39. doi: 10.1186/1471-2350-12-39 (PMC3070627; doi:10.1186/1471-2350-12-39)
Supplement: Additional file 5 — Association of UGRP1 SNPs to Asthma phenotype (Allele based test). Table summarizing the Association of the UGRP1 SNPs calculated using the allele based test for the asthma phenotype. [file 1471-2350-12-39-S5.DOC]

**Supplementary Table 4: Association of UGRP1 SNPs to Asthma phenotype (**Allele based test)

| **SNPs** | **Minor allele** | **Cases**  **(718)** | | **Control**  **(717)** | **Major allele** | **P_trend_** | **OR** | **95%CI** |
| --- | --- | --- | --- | --- | --- | --- | --- | --- |
| rs7726552 | T | | 0.16 | 0.16 | C | 0.9601 | 1.005 | 0.8259-1.223 |
| rs7727031 | C | | 0.05 | 0.06 | A | 0.3042 | 0.8486 | 0.6202-1.161 |
| UGRP1-G-1351A | A | | 0.07 | 0.06 | G | 0.3205 | 1.153 | 0.8706-1.527 |
| rs6882292 | A | | 0.05 | 0.06 | C | 0.3572 | 0.9643 | 0.6334-1.179 |
| rs17107353 | A | | 0.10 | 0.12 | A | 0.1648 | 0.8486 | 0.673-1.07 |
| rs17703574 | T | | 0.04 | 0.05 | C | 0.2162 | 0.8127 | 0.5846-1.13 |
| UGRP1-C1360A | A | | 0.07 | 0.07 | C | 0.4369 | 1.117 | 0.8445-1.479 |
| UGRP1-A1846G | G | | 0.05 | 0.06 | A | 0.4345 | 0.8836 | 0.6478-1.205 |
| rs3910207 | T | | 0.10 | 0.12 | C | 0.1108 | 0.831 | 0.6617-1.044 |
| rs34212847 | A | | 0.07 | 0.07 | C | 0.3348 | 1.147 | 0.8681-1.515 |

P_trend_ is calculated by the Cochran Armitage trend test using the PLINK v 1.06 software.

* P_trend_ < 0.05 for the association test for asthma

OR – Odds ratio; CI – Confidence Interval.
